# Independent Lineage of Lymphocytic Choriomeningitis Virus in Wood Mice (*Apodemus sylvaticus*), Spain

**DOI:** 10.3201/eid1510.090563

**Published:** 2009-10

**Authors:** Juan Ledesma, Cesare Giovanni Fedele, Francisco Carro, Lourdes Lledó, María Paz Sánchez-Seco, Antonio Tenorio, Ramón Casimiro Soriguer, José Vicente Saz, Gerardo Domínguez, María Flora Rosas, Jesús Félix Barandika, María Isabel Gegúndez

**Affiliations:** Universidad de Alcalá, Madrid, Spain (J. Ledesma, L. Lledό, J.V. Saz, M.I. Gegúndez); Instituto de Salud Carlos III, Madrid (C.G. Fedele, M.P. Sánchez-Seco, A. Tenorio); Consejo Superior de Investigaciones Científicas, Seville, Spain (F. Carro, R.C. Soriguer); Consejería de Sanidad y Bienestar Social de la Junta de Castilla y León, Burgos, Spain (G. Domínguez); Centro de Biologia Molecular Severo Ochoa, Madrid (M.F. Rosas); NEIKER-Instituto Vasco de Investigación y Desarrollo Agrario, Vizcaya, Spain (J.F. Barandika)

**Keywords:** Lymphocytic choriomeningitis virus, arenavirus, rodents, Apodemus sylvaticus, Spain, viruses, dispatch

## Abstract

To clarify the presence of lymphocytic choriomeningitis virus (LCMV) in Spain, we examined blood and tissue specimens from 866 small mammals. LCMV RNA was detected in 3 of 694 wood mice (*Apodemus sylvaticus)*. Phylogenetic analyses suggest that the strains constitute a new evolutionary lineage. LCMV antibodies were detected in 4 of 10 rodent species tested.

Lymphocytic choriomeningitis virus (LCMV) is a ubiquitous rodent-borne virus belonging to the family *Arenaviridae*, whose genome consists of 2 single strands of RNA, named small (S) and large (L), respectively. The S segment encodes the nucleocapsid protein (NP) and the glycoprotein precursor (GPC). The L segment encodes a viral RNA-dependent RNA polymerase and a zinc-binding protein. The common house mouse (*Mus musculus)* is the principal reservoir for LCMV. Infected mice can shed the virus in large quantities throughout their lives. Some epidemiologic studies show that ≈9% of wild mice are infected with LCMV (*1*,*2*), and other species of rodents have been reported to be possible reservoirs of LCMV (*2*–*4*).

Humans become infected with LCMV by inhaling contaminated feces or urine, through bite wounds, by vertical route, or after organ transplants (*5*). LCMV is responsible for aseptic meningitis and encephalitis (*6*) and may cause congenital malformations or abortion (*7*). In Spain, 1 case of encephalitis caused by LCMV has been reported (*8*), and recently, LCMV infection has been detected in 4 patients with aseptic meningitis (*9*). LCMV infection in rodents and the general population has also been demonstrated by serologic tests (*2*). The aim of this study was to improve our knowledge of LCMV in rodents in Spain.

## The Study

A total of 866 small mammals were trapped from July 2003 through June 2006 in 19 Spanish provinces. Of those captured, 833 were rodents from 10 species: 694 wood mice (*Apodemus sylvaticus*), 17 yellow-necked mice (*A. flavicollis*), 27 house mice (*M. musculus*), 6 Algerian mice (*M. spretus*), 21 Norway rate (*Rattus norvegicus*), 50 bank voles (*Myodes* [*Clethrionomys*] *glareolus*), 9 snow voles (*Chionomys* [*Microtus*] *nivalis*), 3 Orkney voles (*Microtus arvalis*), 3 Mediterranean pine voles (*Microtus* [*Pitymys*] *duodecimcostatus)*, and 3 garden dormice (*Eliomys quercinus*). Thirty-three were insectivores (18 shrews [*Sorex* spp.] and 15 white-toothed shrews [*Crocidura russula*]). Tissue samples (lungs, kidneys, spleens) were obtained in all cases and stored at –20°C in RNAlater solution (Ambion Inc., Austin, TX, USA) to preserve the RNA and inactivate the virus. Serum samples were only available from 665 specimens.

Serum samples were assayed against LCMV, diluted 1:16 as previously described (*9*), but using immunoglobulins against mice or rats as secondary antibodies. Western blot assays confirmed 25 of the 35 positive serum specimens detected by the immunofluorescence antibody (IFA) assay. The overall prevalence of antibodies against LCMV was 3.76%. Antibodies were detected in 4 species: *A. sylvaticus* (21/536, 3.92%), *M. musculus* (2/24, 8.33%), *M. spretus* (1/6, 16.67%), and *R. norvegicus* (1/21, 4.76%). Titers ranged from 16 to 2,048 by IFA assay.

LCMV-related genome was detected in 3 of 866 specimens corresponding to *A. sylvaticus* mice trapped in Sierra Nevada (SN05), Cabra (CABN), and Grazalema (GR01), 3 well-preserved natural areas in the southern Spain. Only serum specimens from 2 of these rodents were available, and LCMV antibodies were detected in only 1 sample.

Briefly, pools were prepared by mixing 3- to 4-mm pieces of lung, kidney, and spleen from each trapped animal; the mixture was homogenized and their nucleic acid extracted by using RNeasy Mini Kit (QIAGEN, Hilden, Germany) in accordance with the manufacturer’s instructions. The extracted RNA was analyzed by reverse transcription and nested PCR. The first round was performed with primers AREN1+ (5′-_2367_CWATRTANGGCCAICCITCICC_2388_-3′) and AREN1– (5′-_2789_TNRWYAAYCARTTYGGIWCIRTKCC_2813_-3′) and primers AREN2+ (5′-_2396_CANANYTTRTANARNAIRTTYTCRTAIGG_2424_-3′) and AREN2– (5′-_2567_AGYYTNKNNGCNGCIGTIAARGC_2589_-3′) for nested PCR. The symbols + and – correspond to sense and antisense sequences, respectively. Indicated positions correspond to those of LCMV-Armstrong 53b (GenBank accession no. M20869). Primers were designed on conserved motifs of the NP gene and were able to detect arenaviruses from the Old World and from the New World. Amplification products of the expected size (194 bp) were purified and sequenced. Positive results were also obtained when each tissue from these 3 animals was analyzed separately. Viral isolation was not attempted because samples were inactivated with RNA later.

The complete S segment sequence of every detected virus was obtained from lung lysates by using primers designed based on LCMV conserved sequences of the S segments available in GenBank that enable amplification of overlapping complementary DNAs (sequences of the primers are available upon request). The lengths of the S-segments were 3,357, 3,364, and 3,366 nt for samples GR01, SN05, and CABN, respectively (GenBank accession nos. FJ895882–FJ895884, respectively). As expected for LCMV, the sequences defined 2 nonoverlapping genes (genes GPC and NP, with 498 and 558 aa, respectively) arranged in ambisense direction, separated by an intergenic noncoding region, and flanked by 5′ and 3′ ends. Sequence comparison with the complete S segment from other LCMV strains showed deletions and insertions of nucleotides in the noncoding regions (information available on request).

Nucleotide and amino acid sequence distances were calculated by the pairwise distance algorithm (p distance) with MEGA version 3.1 (*10*). Phylograms were reconstructed using the neighbor-joining algorithm and tested with the bootstrap method and 1,000 replicates. GPC gene sequences detected in *A. sylvaticus* mice showed 15.9%–19.7% amino acid differences and 23.4%–27.7% nucleotide differences with the rest of the LCMV sequences (Appendix Table 1). Moreover, *A. sylvaticus-*LCMV sequences of the NP gene differed 8.3%–10.6% at the amino acid level and 19.8%–22.0% at the nucleotide level in comparison with the rest of the LCMV sequences ( Appendix Table 2). Phylogenetic analyses based on the entire amino acid and nucleotide sequences of NP and GPC genes showed that new sequences were grouped with other LCMV strains but in an isolated cluster with a high bootstrap value (Figure).

**Figure:**
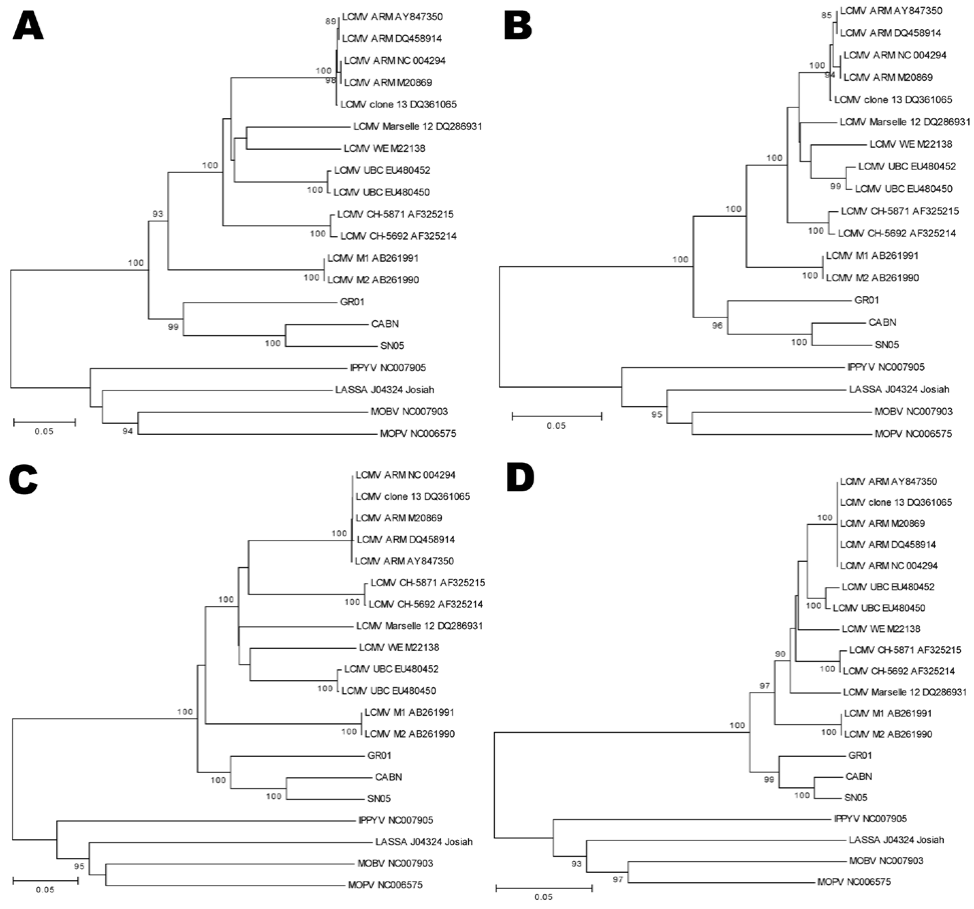
Phylogeny of lymphocytic choriomeningitis virus (LCMV) strains and the viruses detected in this study based on the analysis of complete sequences of amino acids (aa) and nucleotides (nt) of glycoprotein (GPC) and nucleocapsid protein (NP) genes. A) GPC nt; B) GPC aa; C) NP nt; D) NP aa. Each sequence used shows the name of LCMV strain followed by GenBank accession number. Numbers indicate >80% bootstrap values. Scale bars indicate nucleotide substitutions per site. IPPIV, Ippy virus; LASV, Lassa virus; MOBV, Mobala virus; MOPV; Mopeia virus.

## Conclusions

The LCMV seroprevalence detected in this study was similar to that found in other European countries ranging from 3.6% to 16.3% (*3*,*11*,*12*). Specific LCMV antibodies were detected in 4 of 10 rodent species tested; all belonged to the subfamily Murinae and were trapped throughout the country. These results suggest LCMV infection is widespread in Spain.

Phylogenetic analyses showed the close relationship between the new sequences detected in *A. sylvaticus* mice and the previously known LCMV strains, although they formed a separate cluster with a high bootstrap (Figure). The differences found in NP and GPC genes suggest that the new viruses detected in *A. sylvaticus* mice may constitute a new lineage of LCMV. In Lassa virus, similar differences in NP gene sequences served to group different strains into 4 lineages (*13*). Furthermore, comparison of noncoding regions showed that, in spite of the genetic variability in LCMV strains, CABN, GR01 and SN05 had specific deletions and insertions. In conclusion, our data suggest that the described genetic differences of the new sequences contribute to the definition of a new LCMV lineage.

*A. sylvaticus* has previously been related to LCMV (*4*) and its role as a reservoir for this virus has also been suggested (*14*). LCMV genome has recently been detected in this species, but the phylogenetic study grouped the sequence within LCMV strains isolated from *M. musculus* (*15*). By contrast, our analysis showed that CABN, GR01, and SN05 strains define a different branch from the previously known LCMVs, suggesting that *A. sylvaticus* might have been responsible for consolidating genetic changes in these new strains during their evolution, and that *A. sylvaticus* could be their natural reservoir. Further research should be conducted on LCMV in Spain to isolate autochthonous strains and establish their serologic and genomic characterization as well as their potential pathogenicity for humans.

## Supplementary Material

Appendix Table 1 Sequence differences observed between lymphocytic choriomeningitis virus strains and new viruses in rodents by using complete glycoprotein precursor gene sequences, Spain, July 2003-June 2006*dagger

Appendix Table 2 Sequence differences observed between lymphocytic choriomeningitis virus strains and the new viruses by using complete nucleocapsid protein gene sequences, Spain, July 2003-June 2006*dagger
