# Supplementary material for: Independent Lineage of Lymphocytic Choriomeningitis Virus in Wood Mice (Apodemus sylvaticus), Spain
Source: Emerg Infect Dis. 2009 Oct;15(10):1677–80. doi: 10.3201/eid1510.090563 (PMC2866409; doi:10.3201/eid1510.090563)
Supplement: Appendix Table 1 — Sequence differences observed between lymphocytic choriomeningitis virus strains and new viruses in rodents by using complete glycoprotein precursor gene sequences, Spain, July 2003-June 2006*dagger [file 09-0563_appT1-s1.pdf]

Publisher: CDC; Journal: Emerging Infectious Diseases
Article Type: Dispatch; Volume: 15; Issue: 10; Year: 2009; Article ID: 09-0563
DOI: 10.3201/eid1510.090563; TOC Head: Dispatch

Appendix Table 1. Sequence differences observed between lymphocytic choriomeningitis virus strains and new viruses in rodents by using complete glycoprotein precursor gene sequences, Spain, July 2003–June 2006*†

	% Difference															
Strain	CABN	SN05	GR01	ARM AY847350	ARM DQ458914	ARM NC_004294	ARM M20869	Clone 13 DQ361065	Marselle#12 DQ286931	WE M22138	UBC EU480452	UBC EU480450	CH-5871 AF325215	CH-5871 AF325214	M1 AB261991	M1 AB261990
CABN	**–**	**12.6**	**22.9**	**26.5**	**26.5**	**26.7**	**26.7**	**26.7**	**25.5**	**25.6**	**26.9**	**26.7**	**26.4**	**26.7**	**25.2**	**25.2**
SN05	**6.3**	**–**	**22.5**	**27.5**	**27.5**	**27.7**	**27.7**	**27.6**	**24.1**	**26.4**	**27.2**	**27.0**	**26.9**	**26.8**	**25.5**	**25.5**
GR01	**14.8**	**14.8**	**–**	**24.6**	**24.6**	**24.8**	**24.8**	**24.7**	**23.4**	**23.8**	**24.8**	**24.9**	**25.0**	**24.8**	**24.5**	**24.5**
ARM AY847350	**17.8**	**18.2**	**16.5**	–	0	0.4	0.4	0.3	16.0	15.0	14.6	14.5	15.8	16.0	21.9	21.9
ARM DQ458914	**17.8**	**18.2**	**16.5**	0	–	0.4	0.4	0.3	16.0	15.0	14.6	14.5	15.8	16.0	21.9	21.9
ARM NC_004294	**18.0**	**18.4**	**16.7**	0.6	0.6	–	0	0.4	16.3	15.0	14.8	14.6	15.9	16.2	22.1	22.1
ARM M20869	**18.0**	**18.4**	**16.7**	0.6	0.6	0	–	0.4	16.3	15.0	14.8	14.6	15.9	16.2	22.1	22.1
Clone 13 DQ361065	**17.8**	**18.2**	**16.3**	0.4	0.4	0.6	0.6	–	16.0	14.9	14.5	14.3	15.7	16.0	21.7	21.7
Marselle#12 DQ286931	**18.2**	**18.2**	**17.8**	4.2	4.2	4.4	4.4	3.8	–	14.1	15.1	15.1	16.2	16.4	21.2	21.2
WE M22138	**19.5**	**19.7**	**17.1**	5.9	5.9	5.9	5.9	5.5	5.9	–	14.0	14.1	17.2	17.3	22.2	22.2
UBC EU480452	**18.4**	**19.0**	**17.3**	5.5	5.5	5.7	5.7	5.1	4.9	5.3	–	0.5	14.8	15.0	22.1	22.1
UBC EU480450	**18.0**	**19.0**	**17.5**	5.1	5.1	5.3	5.3	4.7	4.9	5.7	0.8	–	15.0	15.1	22.2	22.2
CH-5871 AF325215	**17.3**	**17.8**	**18.0**	5.5	5.5	5.7	5.7	5.3	5.5	8.0	6.3	6.3	–	0.8	21.2	21.2
CH-5871 AF325214	**17.3**	**17.8**	**18.0**	5.3	5.3	5.5	5.5	5.1	5.3	7.8	6.1	6.1	0.8	–	21.5	21.5
M1 AB261991	**16.7**	**16.7**	**15.9**	9.5	9.5	9.7	9.7	9.1	9.3	11.2	9.7	9.5	9.1	9.1	–	0
M1 AB261990	**16.7**	**16.7**	**15.9**	9.5	9.5	9.7	9.7	9.1	9.3	11.2	9.7	9.5	9.1	9.1	0	–

*Values above the diagonal are % nucleotide differences, and values below the diagonal are % amino acid differences. Differences between CABN, GR01, and SN05 compared with others are shown in **boldface.**
†Values of nucleotide and amino acid differences were calculated by p distance and multiplied by 100.
