# Supplementary material for: Independent Lineage of Lymphocytic Choriomeningitis Virus in Wood Mice (Apodemus sylvaticus), Spain
Source: Emerg Infect Dis. 2009 Oct;15(10):1677–80. doi: 10.3201/eid1510.090563 (PMC2866409; doi:10.3201/eid1510.090563)
Supplement: Appendix Table 2 — Sequence differences observed between lymphocytic choriomeningitis virus strains and the new viruses by using complete nucleocapsid protein gene sequences, Spain, July 2003-June 2006*dagger [file 09-0563_appT2-s2.pdf]

Publisher: CDC; Journal: Emerging Infectious Diseases
Article Type: Dispatch; Volume: 15; Issue: 10; Year: 2009; Article ID: 09-0563
DOI: 10.3201/eid1510.090563; TOC Head: Dispatch

Appendix Table 2. Sequence differences observed between lymphocytic choriomeningitis virus strains and the new viruses by using complete nucleocapsid protein gene sequences, Spain, July 2003–June 2006*†

	% Difference															
Strain	CABN	SN05	GR01	ARM AY847350	ARM DQ458914	ARM NC_004294	ARM M20869	Clone 13 DQ361065	Marselle#12 DQ286931	WE M22138	UBC EU480452	UBC EU480450	CH-5871 AF325215	CH-5871 AF325214	M1 AB261991	M1 AB261990
CABN	**–**	**11.3**	**18.6**	**21.1**	**21.0**	**21.0**	**21.0**	**21.0**	**21.4**	**20.9**	**22.0**	**21.8**	**21.1**	**21.0**	**21.9**	**21.9**
SN05	**2.9**	**–**	**17.1**	**20.4**	**20.2**	**20.4**	**20.4**	**20.4**	**21.1**	**21.1**	**21.6**	**21.4**	**21.3**	**21.2**	**21.7**	**21.7**
GR01	**7.0**	**6.5**	**–**	**20.8**	**20.9**	**20.8**	**20.8**	**20.8**	**19.8**	**20.7**	**20.7**	**20.5**	**20.3**	**20.3**	**21.5**	**21.5**
ARM AY847350	**9.4**	**9.2**	**9.7**	–	0.2	0.1	0.1	0.1	14.9	15.4	14.1	14.0	14.7	14.6	19.6	19.6
ARM DQ458914	**9.4**	**9.2**	**9.7**	0	–	0.1	0.1	0.1	15.1	15.4	14.2	14.1	14.9	14.8	19.6	19.6
ARM NC_004294	**9.4**	**9.2**	**9.7**	0	0	–	0	0	15.0	15.5	14.2	14.1	14.8	14.7	19.6	19.6
ARM M20869	**9.4**	**9.2**	**9.7**	0	0	0	–	0	15.0	15.5	14.2	14.1	14.8	14.7	19.6	19.6
Clone 13 DQ361065	**9.4**	**9.2**	**9.7**	0	0	0	0	–	15.0	15.5	14.2	14.1	14.8	14.7	19.6	19.6
Marselle#12 DQ286931	**9.9**	**9.6**	**9.6**	5.1	5.1	5.1	5.1	5.1	–	15.1	14.5	14.4	16.7	16.6	19.6	19.6
WE M22138	**9.4**	**9.9**	**10.1**	4.2	4.2	4.2	4.2	4.2	5.2	–	13.2	13.1	15.3	15.1	19.5	19.5
UBC EU480452	**8.8**	**9.2**	**9.4**	3.2	3.2	3.2	3.2	3.2	4.9	4.3	–	0.4	15.6	15.5	19.3	19.3
UBC EU480450	**8.3**	**8.7**	**8.8**	2.7	2.7	2.7	2.7	2.7	4.5	4.0	0.9	–	15.4	15.3	19.1	19.1
CH-5871 AF325215	**9.6**	**9.6**	**9.6**	4.9	4.9	4.9	4.9	4.9	5.8	4.9	4.9	4.3	–	0.3	21.1	21.1
CH-5871 AF325214	**9.2**	**9.2**	**9.2**	4.5	4.5	4.5	4.5	4.5	5.4	4.5	4.5	4.0	0.4	–	21.0	21.0
M1 AB261991	**10.5**	**10.6**	**9.7**	6.7	6.7	6.7	6.7	6.7	7.0	6.7	6.7	6.3	7.4	7.0	–	0
M1 AB261990	**10.5**	**10.6**	**9.7**	6.7	6.7	6.7	6.7	6.7	7.0	6.7	6.7	6.3	7.4	7.0	0	–

*Values above the diagonal are % nucleotide differences and values below the diagonal are % amino acid differences. Differences between CABN, GR01, and SN05 compared with others are shown in **boldface.**
†Values of nucleotide and amino acid differences were calculated by p distance and multiplied by 100.
